# Unveiling the Metabolomic Profile of Oily Sensitive Skin: A Non-Invasive Approach

**DOI:** 10.3390/ijms252011033

**Published:** 2024-10-14

**Authors:** Jiaqi Zhang, Fan Wu, Jun Wang, Yi Qin, Yao Pan

**Affiliations:** Department of Cosmetics, School of Light Industry Science and Engineering, Beijing Technology and Business University, Beijing 100048, China; 2230402137@st.btbu.edu.cn (J.Z.); 2230401031@st.btbu.edu.cn (F.W.); 2330402112@st.btbu.edu.cn (J.W.); 2330402109@st.btbu.edu.cn (Y.Q.)

**Keywords:** compromised skin barrier, untargeted metabolomics, oily sensitive skin, questionnaire

## Abstract

Skin barrier impairment is becoming increasingly common due to changes in lifestyle and modern living environments. Oily sensitive skin (OSS) is a condition that is characterized by an impaired skin barrier. Thus, examining the differences between OSS and healthy skin will enable a more objective evaluation of the characteristics of OSS and facilitate investigations of potential treatments. Initially, a self-assessment questionnaire was used to identify patients with OSS. Biophysical measurements and LAST scores were used to determine whether skin barrier function was impaired. Epidermal biophysical properties, including skin hydration, transepidermal water loss (TEWL), sebum content, erythema index (EI), and a* value, were measured with noninvasive instruments. We subsequently devised a noninvasive D-square sampling technique to identify changes in the skin metabolome in conjunction with an untargeted metabolomics analysis with an Orbitrap Q ExactiveTM series mass spectrometer. In the stratum corneum of 47 subjects, 516 skin metabolites were identified. In subjects with OSS, there was an increase in the abundance of 15 metabolites and a decrease in the abundance of 48 metabolites. The participants with OSS were found to have the greatest disruptions in sphingolipid and amino acid metabolism. The results revealed that an impaired skin barrier is present in patients with OSS and offers a molecular target for screening for skin barrier damage.

## 1. Introduction

One widespread and complicated issue that impacts a considerable proportion of the world’s population is an impaired skin barrier [1]. This condition includes a broad spectrum of skin types, such as dry sensitive skin, oily acne, and oily sensitive skin (OSS) [2]. When exposed to external stimuli, including allergies, irritants, and other external conditions, these various skin types commonly exhibit distinct symptoms and reactions [3]. The root cause of an impaired skin barrier is a breakdown in the protective function of the skin, leading to increased transepidermal water loss (TEWL), modified lipid composition, and heightened vulnerability to infection and inflammation [4,5]. People with compromised skin barriers, especially those with OSS, may suffer from a variety of conditions that negatively impact their quality of life, including social distress, nervousness, and depression [6,7].

The field of metabolomics is a relatively new research field that aims to identify and quantify all metabolites in biological systems to analyze the overall metabolic profile of individual cells, tissues, organs, or even entire organisms [8,9]. The majority of these metabolites are naturally occurring tiny molecules with a relative molecular mass of less than 1000 [10]. For example, the clinical severity of recessive dystrophic epidermolysis bullosa was shown to be correlated with reductions in serine, lysine, tryptophan, tyrosine, arginine, histidine, glutamine, and proline levels [11]. The changes in serum metagenomic features between individuals with psoriasis and healthy controls were examined [12]. The findings revealed that individuals with psoriasis had higher levels of amino acids, lactic acid, and urea and lower levels of crotonic acid and azelaic acid than healthy people did [13]. These results contribute to the understanding of psoriasis pathophysiology and the search for putative biomarkers. A nontargeted approach was used to examine the organic metabolites that accumulate in the skin and to contrast the volatile metabolomic features of melanoma with those of normal skin [14]. The findings of these analyses demonstrated that melanoma patients had noticeably higher amounts of lauric and palmitic acids [15]. To a certain extent, skin metabolites can be used as indicators of skin health [16]. The use of metabolomics technology to analyze skin metabolites can advance our knowledge of the skin and certain skin disorders while also strengthening the theoretical foundations of skin care and disease management [17].

In the realm of dermatology, one of the most prevalent complaints is oily skin. Seborrheic dermatitis, acne, and enlarged pores are typically present in conjunction with this condition. Furthermore, greasy skin might negatively impact self-perception [18]. Moreover, the condition known as sensitive skin is characterized by subjective hyperreactivity to external stimuli [19]. When these two conditions are combined, OSS becomes very uncomfortable and resistant to therapy [20]. Metabolic methods precisely detect potential variations in skin features. Therefore, the aim of our study was to explore the differences in skin biophysical properties and corneocyte metabolism between OSS and healthy skin (HS). In the present work, a noninvasive sampling technique was performed, and an untargeted metabolomics screening technique was developed to investigate the possible consequences of OSS at the omics level [21]. The results revealed metabolomic alterations in the facial skin of the subjects.

## 2. Results

### 2.1. Subject Skin Type Categorization Basis of the OSS Self-Assessment Scale

The subjects were divided into four groups on the basis of the self-assessment scale they completed, which was examined in two dimensions. The groups included the HS group, the mild OSS group, the moderate OSS group, and the severe OSS group. On the basis of the thresholds of the OSS self-assessment scale, we grouped the subjects by analyzing both the sensitive and oily scores in combination. Table 1 shows the group numbers, the mean ages of the subjects, and the scale scores of the different groups. Among the 47 participants, the average age was 32.55 years, and there was no discernible age difference among the four groups. A total of 11 participants were included in the severe OSS group, and 12 individuals were included in each of the other three groups. The OSS group’s scores for both sensitivity and oiliness were significantly greater than those of the healthy group, and the scores increased with the degree of oil sensitization (*p* < 0.001).

### 2.2. Variations in the Biophysical Characteristics and Lactic Acid Stinging Test (LAST) Scores of the OSS and HS Groups

**Figure 1:**
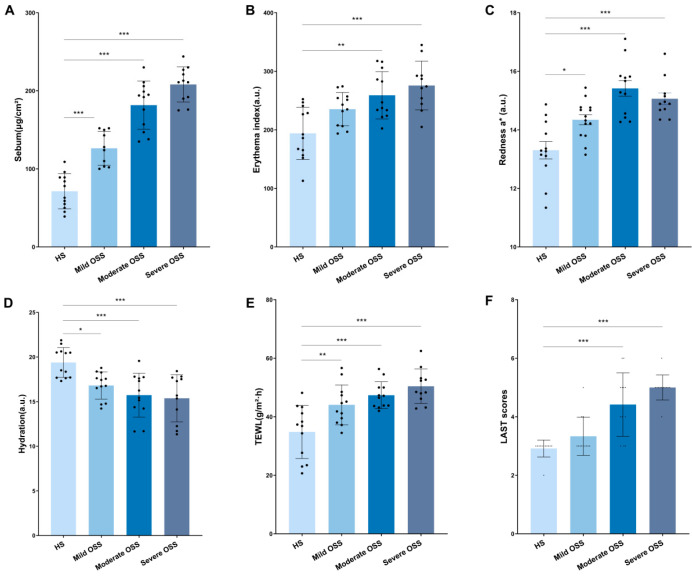
The results in healthy skin (HS), mild oily sensitive skin (mild OSS), moderate oily sensitive skin (moderate OSS), and severe oily sensitive skin (severe OSS) subjects of sebum content in ug/cm^2^ (**A**), erythema index in arbitrary units (a.u.) (**B**), redness a* values in arbitrary units (a.u.) (**C**), skin hydration in arbitrary units (a.u.) (**D**), and transepidermal water loss (TEWL) in g/m^2^∙h (**E**), LAST scores (**F**). The values are expressed as mean ± SD. * *p* < 0.05, ** *p* < 0.01, *** *p* < 0.001 compared with healthy skin (*n* = 47).

Figure 1 illustrates the notable variations in the LAST scores and biophysical characteristics among the four separate groups. There were increasing trends in individual sebum levels, erythema index (EI), a* values, TEWL, and LAST scores, whereas hydration decreased. This study revealed a statistically significant increase in sebum production across the mild, moderate, and severe OSS groups and the HS group (*p* < 0.001) (Figure 1A). Furthermore, the subjects in the moderate OSS group presented significantly greater EI values than those in the HS group (*p* < 0.01), and there was also a statistically significant increase in the EI values in the severe OSS group compared to the HS group (*p* < 0.001) (Figure 1B). In addition, the a* values of the subjects in the mild OSS group were significantly greater than those in the HS group (*p* < 0.05), and the a* values of the moderate and severe OSS groups were significantly greater than those in the HS group (*p* < 0.001) (Figure 1C). In addition, the mild OSS group showed significantly lower hydration than the HS group (*p* < 0.05), and there was a statistically significant decrease in hydration between the moderate and severe OSS groups and the HS group (*p* < 0.001) (Figure 1D). As shown in Figure 1E, there was a statistically significant increase in TEWL in the moderate and severe OSS groups compared with the HS group (*p* < 0.001), and the mild OSS group also had significantly greater TEWL (*p* < 0.01). Additionally, there were statistically significant differences in the LAST scores between the HS group and the moderate and severe OSS groups (*p* < 0.001) (Figure 1F). Significant differences were observed in both semisubjective and objective indicators across the four groups in our investigation. In general, the results of the subjective and objective indicators revealed that as the degree of skin oil sensitivity increased, the sebum levels, EI, a* values, TEWL and LAST scores significantly increased, and skin hydration significantly decreased, which suggests that OSS exhibits obvious barrier damage.

### 2.3. Comprehensive Analysis of Skin Metabolites via the PLS-DA Approach for Metabolite Classification

**Figure 2:**
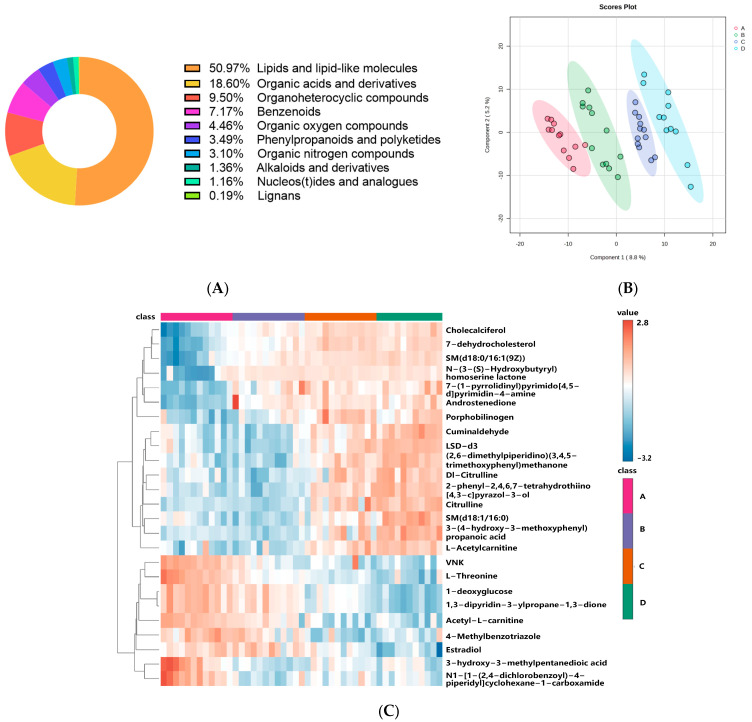
Multivariate analysis of skin metabolites. (**A**) Distribution of various skin metabolites. (**B**) PLS-DA score plots among healthy skin (HS) group, mild oily sensitive skin group (mild OSS), moderate oily sensitive skin group (moderate OSS), severe oily sensitive skin (severe OSS) group. (**C**) The hierarchical clustering heatmaps of metabolites with differences among four groups. Each colored cell on the map corresponds to peak intensity, with samples shown in column and metabolites shown in row. A, HS group, B, mild OSS group, C, moderate OSS group, D, severe OSS group. (For interpretation of the references to color in this figure legend).

A total of 516 putatively annotated metabolites were identified among the 887 metabolites that were categorized into four groups (Table S1). The metabolites were categorized into 10 superclass groups according to the HMDB: lipids and lipid-like molecules, organic acids and derivatives, organoheterocyclic compounds, benzenoids, organic oxygen compounds, phenylpropanoids and polyketides, organic nitrogen compounds, alkaloids and derivatives, nucleosides/nucleotides and analogs, and lignans, neolignans, and related compounds (Figure 2A). With the exception of lignans, neolignans, and related compounds, the other nine categories were discovered in the positive mode, but two categories (organic nitrogen compounds and alkaloids and their derivatives) were not detected in the negative mode. A total of 50.97% (263/516) of the metabolites in the skin were lipids or lipid-like compounds, indicating that these were the predominant skin metabolites. Organic acids and their derivatives were the second most prevalent type of metabolite in the skin, accounting for 18.60% (96/516) of the metabolites.

For multivariate data analysis, the partial least squares discriminant analysis (PLS-DA) model was employed. Figure 2B shows the model score plot. The PLS-DA models were validated using two indicators: predictive capacity (Q2) and goodness-of-fit (R2Y). The results revealed that the four distinct groups of skin metabolites differed significantly from one another (R2Y, Q2 > 0.5; Figure S1). The distribution of metabolites at various levels in the OSS groups and in the healthy control group is displayed via a heatmap. The top 25 compounds were selected for presentation on the basis of their VIP values (Figure S2), as indicated in Figure 2C. These findings demonstrated that the differences in skin metabolites among the four skin type groups were significant.

**Figure 3:**
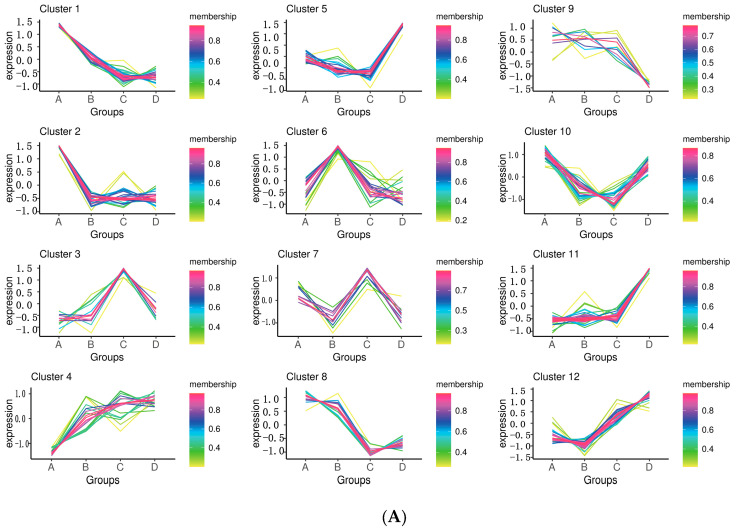

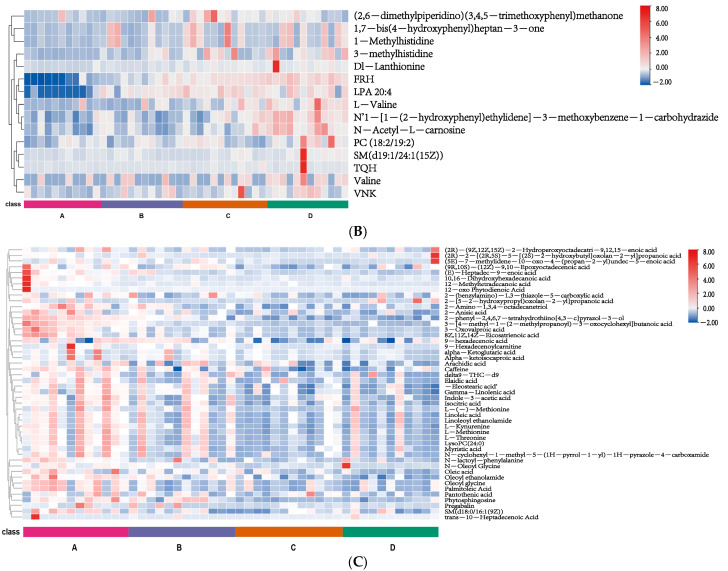
Clustering metabolites using k-means. (**A**) The clustering algorithm results from k-means method. (**B**) The hierarchical clustering heatmaps of increased metabolites with increasing skin oil sensitivity. (**C**) The hierarchical clustering heatmaps of decreased metabolites with increasing skin oil sensitivity. A, healthy skin group, B, mild oily sensitive skin group, C, moderate oily sensitive skin group, D, severe oily sensitive skin group. Each colored cell on the map corresponds to peak intensity, with samples shown in column and metabolites shown in row.

The k-means method was used to cluster the metabolites across the four groups (Figure 3A), and the results revealed that as oily skin sensitivity increased, 15 metabolite response values significantly increased, and 48 significantly compared with these levels in the HS group (Tables S2 and S3) (Figure 3B,C). The algorithm results revealed that metabolites regularly increase or decrease as skin oil sensitivity increases. It can also be assumed that metabolite levels increase or decrease with increasing skin barrier permeability, indicating that metabolomics is a practical and useful technique for evaluating skin health.

### 2.4. Insights from PLS-DA Models and Volcano Plot Analysis Based on Changes in Metabolite Levels in the OSS and HS Groups

The results of the volcano plots also revealed that skin metabolism could be affected by different levels of skin oil sensitivity (VIP > 1, *p*-value < 0.05, fold change (FC) ≥ 2 or FC ≤ 0.5; typical metabolic volcano plots, PLS-DA plots, and validation plots are displayed in Figures S3–S6). An overview of the differential abundance of metabolites is shown in Table 2.

The distribution of metabolites in the HS group could be distinguished from that in the groups with moderate and severe OSS using the PLS-DA models (R2Y, Q2 > 0.5; Figures S3 and S4).

Thirty-three metabolites were identified as common annotated differentially abundant metabolites via volcano plots. These included 22 lipids and lipid-like molecules, 6 organic acids and derivatives, 2 organoheterocyclic compounds, 2 benzenoids, and 1 organic oxygen compound. For example, in individuals with moderate and severe OSS, the intensity of SM(d18:1/16:0), a characteristic phosphosphingolipid, was reduced by 67.83% and 99.9%, respectively, in comparison with that of the metabolites of healthy skin. In the HS and moderate OSS groups, the SM(d18:1/16:0) AUC and VIP values were 1.00 and 3.23, respectively. The AUC and VIP of SM(d18:1/16:0) were 1.00 and 3.83, respectively, between the healthy and severe OSS groups, indicating a substantial reduction.

**Figure 4:**
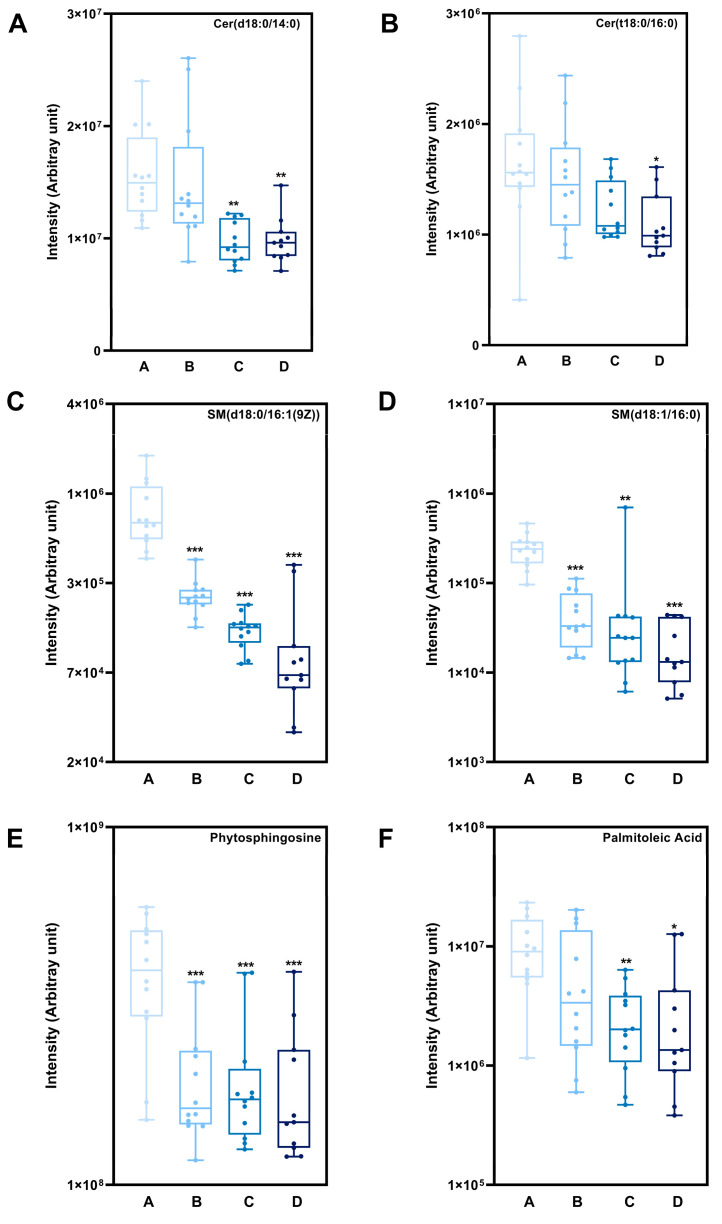
Intensity of two CERs, two SMs, a sphingosine, and a fatty acid with significant difference induced by impaired skin barrier. (**A**) Cer(d18:0/14:0), (**B**) Cer(t18:0/16:0), (**C**) SM(d18:0/16:1(9Z)), (**D**) SM(d18:1/16:0), (**E**) Phytosphingosine, (**F**) Palmitoleic acid. * *p* < 0.05, ** *p* < 0.01, *** *p* < 0.001 compared with healthy skin. A, healthy skin group, B, mild oily sensitive skin group, C, moderate oily sensitive skin group, D, severe oily sensitive skin group.

In addition, we compared the statistically significant differences between the four groups in terms of the intensities of the two CERs, two SMs, a sphingosine, and a fatty acid. For Cer(d18:0/14:0), the mild and severe OSS groups presented significantly lower intensities than the healthy skin group (*p* < 0.01) (Figure 4). For Cer(t18:0/16:0), only the severe OSS group presented a significantly lower intensity than healthy skin did (*p* < 0.05) (Figure 4). For SM(d18:0/16:1(9Z)), subjects with mild, moderate, and severe OSS had considerably lower intensities than those with HS (*p* < 0.001). For SM(d18:1/16:0), the mild and severe OSS groups presented significantly lower intensities (*p* < 0.001), and the moderate OSS group presented a significantly lower intensity than the healthy skin group (*p* < 0.01) (Figure 4). For phytosphingosine (sphingosine), subjects with mild, moderate, and severe OSS had considerably lower intensities than those with HS (*p* < 0.001). For palmitoleic acid (fatty acid), the moderate OSS groups presented significantly lower intensities (*p* < 0.01), and the severe OSS group presented a lower intensity than that did the healthy skin group (*p* < 0.05) (Figure 4). As a result, all individuals with OSS showed substantial down regulation of total SM, and the intensity of SM decreased as the skin became more susceptible to skin barrier impairment.

The distribution of metabolites in the mild, moderate, and severe OSS groups could be distinguished via PLS-DA models (R2Y, Q2 > 0.5; Figures S5 and S6). Six metabolites were identified as common, annotated differentially, abundant metabolites on the basis of the volcano plots. These metabolites included three lipids and lipid-like molecules, one organic acid and derivative, one organic nitrogen compound, and one organic oxygen compound. For example, in individuals with moderate and severe OSS, the intensity of gluconolactone, a characteristic organic oxygen compound, increased by 57.07% and 99.9%, respectively, compared with that of mild OSS metabolites. In the mild and moderate OSS groups, the gluconolactone AUC and VIP values were 0.76 and 1.46, respectively. The AUC and VIP values for gluconolactone were 0.80 and 1.32, respectively, between the mild and severe OSS groups, indicating a substantial increase.

## 3. Discussion

### 3.1. Variation in the Subjective and Objective Indicators Induced by Skin Barrier Impairment

Clinical investigations have indicated that those with sensitive skin might have a deficient epidermal permeability barrier and insufficient hydration [22]. Furthermore, previous research has shown that human sebum may have a deleterious effect on the structural and functional features of the barrier, increasing skin permeability [23]. The traits of both oily and sensitive skin are present in patients with OSS. In recent years, several skin self-assessment scales have been created for the skin abnormalities of patients [24,25]. For this study, our scale was split into two sections: one asked about levels of facial sensitivity, and the other asked about the degree to which a face was oily [26]. The results of this study show that our scale can be used to scientifically and effectively differentiate between HS and different degrees of OSS.

Our results revealed a substantial increase in the sebum content, EI, a* values, TEWL, and LAST scores in the faces of participants in the OSS group compared with those in the HS group, whereas their hydration decreased (Figure 1). Dermal function tests, which involve various noninvasive testing techniques to probe skin sensitivity, including sebum, color, TEWL, or hydration, were created as objective alternatives to neurosensory testing [27]. In addition, a higher LAST score could indicate more sensitive skin due to greater lactic acid penetration [28]. TEWL is often instantaneously measured to evaluate barrier function [29]. Most studies have reported greater TEWL in the unchallenged skin of subjects with sensitive skin than in those with nonsensitive skin [30,31,32,33] and in LAST stingers than in nonstingers [34,35]. In addition, a previous study revealed that the group with sensitive skin had more sebum on their foreheads, considerably higher a* values, and a greater EI on their cheeks than the HS group did [36]. Notably, Asian patients, who report sensitive skin more frequently than Caucasian patients do, have clinically oilier skin [37,38]. This also explains our findings, which revealed a positive correlation between individuals’ TEWL and sebum levels and considerably greater LAST scores in the OSS group than in the HS group (Figure 1). These findings suggest that individuals with increased skin oil sensitivity may have impaired barrier function and may trigger inflammatory reactions.

### 3.2. Variations in the Metabolome of the OSS and HS Groups

The top 25 compounds were indicated in Figure 2C for presentation on the basis of their VIP values; cholecalciferol (vitamin D3) and 7-dehydrocholesterol are two metabolites with the highest VIP scores. Depending on the UV light intensity, 7-dehydrocholesterol produces vitamin D, which is then hydroxylated to 25 hydroxyvitamin D3 (25(OH)D) and then further hydroxylated to 1,25 dihydroxyvitamin D3 (calcitriol) [39]. It is also widely known that calcitriol strictly regulates the processes of keratinocyte differentiation and proliferation [40]. The current study’s results showed substantial differences in cholecalciferol and 7-dehydrocholesterol (Figure 1C), with larger concentrations of both compounds in OSS. These findings may point to a reduction in converting to calcitriol, which might cause aberrant keratinocyte proliferation and differentiation.

The results of the volcano plots suggested that skin metabolism could be affected by different levels of skin oil sensitivity (VIP > 1, *p*-value < 0.05, fold change (FC) ≥ 2 or FC ≥ 0.5; typical metabolic volcano plots, PLS-DA plots, and validation plots are displayed in Figures S3–S6). An overview of the differential metabolomes is shown in Table 2.

#### 3.2.1. Sphingolipid Metabolism

For skin barrier testing, the combination of D-square with UHPLC-MS/MS is more appropriate since it may reduce the detection time and be gentler for the subjects. Using the D-square sampling approach in conjunction with UHPLC–Orbitrap–HRMS analysis, 356 metabolites were identified in a previous work [21]. However, 516 metabolites were identified in the present study. This difference might be due to the difference in sample collection methods; we used ten successive layers of tape on the individuals’ facial cheeks, whereas the previous study used only four layers, which could be the primary cause for the difference in the number of metabolites detected. According to a study that identified facial sebum components of sensitive and healthy skin, a deficiency in water and excessive apoptosis may be the primary causes of barrier dysfunction in patients with sensitive skin [41]. This result is consistent with our findings, and the D-square method was utilized to move the layers of corneocytes to assess the compromised skin barrier. This suggests that the approach used in this study can be used to effectively screen a larger number of metabolites and is a practical technique for evaluating the skin barrier.

As the primary constituent of the skin barrier, skin lipids are essential for preserving skin health. Skin lipids consist of the lipids that are released by sebaceous glands, the lipids that are created during keratinocyte metabolism, and the lipids that are metabolized by microorganisms on the surface of the skin [42]. Corneocytes are embedded in an extracellular, highly organized lipid matrix of hydrophobic lipids consisting of approximately 50% ceramides (CERs), 25% cholesterol, and 15% long- and extremely long-chain fatty acids [43]. The most important lipids for the epidermal barrier are ceramides [44]. The primary probarrier precursor lipids, sphingomyelins (SMs), are packaged in lamellar bodies combined with hydrolytic enzymes and secreted into the intercellular region between the stratum corneum and stratum granulosum. Sphingomyelin synthase (SMS) is an enzyme that generates SM from CER and phosphatidylcholine [45]. SM in the epidermis is a precursor to CER [46], and SM and CER are both pivotal components of sphingolipids. In this study, impaired barrier functions and significantly lower SM and CER levels in the stratum corneum have been observed in the skin of subjects with OSS compared with those with HS (Figure 4). Hence, it could be inferred that SM and CER can serve as reliable molecular targets for mechanistic screening of impaired skin barriers. The imbalance in atopic skin is coupled with a severe lack of lipid components, leading to insufficient synthesis of the protective lamellar barrier. Furthermore, a deficiency of sphingolipids in the stratum corneum might be a key physiologic element in barrier-disrupted skin [47].

The human skin barrier needs epidermal homeostasis, which includes keratinocyte proliferation, migration, and differentiation [48]. Ultraviolet (UV) radiation may have increased the breakdown of the skin barrier [49]. The current investigation was conducted to assess metabolic alterations in the skin keratinocytes of naked rats exposed to UV radiation [50]. Consistent with our findings, the previous results demonstrated that UV radiation produces changes in the phospholipid profile of keratinocytes, particularly via the suppression of SM. Furthermore, the results revealed that the function of lipid domains in keratinocytes is dependent on SM [51]. When replicative senescence occurs in keratinocytes, the quantity of SM-rich domains decreases, which is accompanied by reduced SM detection [52].

We also compared the changes in sphingosine and fatty acids among the declining chemicals (Figure 3C). It is known that sphingosine, fatty acids, phosphoric acid, and nitrogenous bases comprise SM [53]. As we previously mentioned, the major SC lipids, fatty acids, and SMs have distinct roles in maintaining normal barrier functions [54]. CERs were demonstrated to have an influence on (and are influenced by) the integrity of the SC, whereas free fatty acids have a major role in lipid bilayer formation and pH maintenance [55]. The results also indicated that the drop in sphingosine may result in decreased ceramide content in the skin. As a result, we found significant declines in such substances, which supports the notion that metabolic disorganization occurs in the OSS for all types of lipids.

Phospholipids are abundant in epidermal cells, particularly keratinocytes, and they not only aid in creating and maintaining the epidermal barrier but also play a crucial role in cell metabolism [56]. A prior study revealed dysregulated sphingolipid metabolism in skin samples from a rat model of acne [57]. Moreover, a previous investigation revealed that the sphingolipid signaling pathway was the most strongly affected pathway in the plasma of patients with moderate-to-severe acne [58]. Sphingolipid metabolites are potential signaling molecules that alter cellular processes in animals and are associated with skin barrier function impairments [59]. A previous study revealed that changes in sphingolipid metabolism might also indicate epidermal barrier degradation [60]. Hence, in our study, sphingolipid metabolism might have changed the function of corneocytes, which is linked to a compromised skin barrier.

#### 3.2.2. Amino Acid Metabolism

The k-means method was used to screen 11 amino acids for the category of annotated common metabolites (Tables S2 and S3) (Figure 3B,C). Five amino acids were found to be elevated (1-methylhistidine, 3-methylhistidine, Dl-lanthionine, L-valine, and valine), and six amino acids were found to be decreased (L-(-)-methionine, L-methionine, L-threonine, N-lactoyl-phenylalanine, oleoyl glycine, and pregabalin).

1-1-Methylhistidine and 3-methylhistidine are the two main L-histidine (HIS) derivatives found in human tissue and are also two metabolites that were significantly increased in this study [61]. The conversion of histidine to carbosine or histamine, metabolic products with functions in oxidative stress, cognitive function, gastric secretions, allergy reactions, and cardiometabolic health, may be crucial for these roles [62]. Considering that inflammatory skin also has significantly higher histamine levels, it is likely that histamine contributes to the pathophysiology of this condition [63]. Inflammation increases the sensitivity of the skin, which can cause itching, dryness, and redness [64]. As a result, the earliest biological reactions, such as erythema and skin redness, are the most evident signs of inflammation [65]. Our study also revealed that, compared with the HS group, the OSS group had higher EI and a* values and that these values increased as the degree of oil sensitivity increased (Figure 1). This is also indicative of certain inflammatory signs in OSS, and the high histidine level suggests that the skin is still inflamed in this particular population. The findings of this study demonstrated that the amount of histidine increased in proportion to the degree of oil sensitivity of the skin, indicating that patients with OSS are more prone to inflammation, which is consistent with the features of OSS reported in other studies.

L-(-)-Methionine and L-methionine are two metabolites that are typically significantly decreased, and it is well-established that reduced levels of methionine and its metabolites are associated with systemic impairment of cellular function in several organs [66]. Hepatocytes may also easily use methionine to directly produce the low-molecular-weight antioxidant glutathione [67]. Methionine, on the other hand, has been demonstrated to chelate lead and eliminate it from tissues, reducing oxidative stress [68]. Therefore, overproduction of ROS caused by an impaired skin barrier damages DNA and membrane phospholipids, leading to cell death, tissue damage, persistent inflammatory reactions, and fibrogenesis [69].

Notably, N-lactoyl-phenylalanine is essential for the synthesis of melanin, which may indicate hyperpigmentation in OSS [70]. Melanocytes exert an immunomodulatory effect through melanin biosynthesis, which reduces the production of proinflammatory cytokines by interacting with T lymphocytes and fibroblasts. Furthermore, melanocytes respond to external factors by releasing immunomodulators, such as inducible nitric oxide synthase and proinflammatory cytokines, influencing nearby cells, such as corneocytes and fibroblasts, and activating lymphocytes. The variations in this amino acid observed in this study might be associated with the combined effects of hypersensitive and oily skin [71]. Our findings are also consistent with previous research that demonstrated a decrease in phenylalanine levels due to aberrant melanin deposition and excessive synthesis triggered by the inflammatory process of acne, resulting in pigment sequelae.

## 4. Materials and Methods

### 4.1. Subjects

The principles outlined in the Declaration of Helsinki were adhered to throughout the course of this inquiry. Beijing Technology and Business University’s Ethics Committee Board gave its approval of the study procedure. Every participant provided written informed permission. After enrolling, each participant filled out a scale that we had previously developed for the assessment of OSS, and subjects were categorized into four groups: the HS group, the mild OSS group, the moderate OSS group, and the severe OSS group [25].

Chinese female participants in good health between the ages of 25 and 40 met the inclusion criteria. The following were the exclusion criteria: (i) individuals with any facial skin diseases (e.g., acne, allergic dermatitis, glucocorticoid-dependent dermatitis, rosacea, infections), systemic disorders and ongoing pharmacological treatment, (ii) extensive exposure to sunlight or artificial ultraviolet rays within the previous month, (iii) physical/chemical treatments such as laser and chemical exfoliation performed on the face in the past 3 months, (iv) pregnancy, breastfeeding, or recent intention to conceive, (v) severe systemic diseases, immune deficiencies, or autoimmune diseases, (vi) individuals with an allergic predisposition, (vii) experts believe that there are other iatrogenic reasons that may affect the participant’s test results, (viii) inability to understand and/or answer the questionnaire, and (ix) individuals who refused to participate.

### 4.2. Lactic Acid Stinging Test

A 10% concentration of lactic acid (Sigma Aldrich, St. Louis, MO, USA) was created in distilled water. One side of the nasolabial fold received a 10% lactic acid solution, while the other was randomly given a saline solution as a placebo. In a single sheet of filter paper (8 mm in diameter), 50 μL of solution was absorbed, with a light stroke of the solution applied to each side. The individuals were assessed before applying 10% lactic acid or placebo solutions, as well as 2.5 and 5 min after. The test subjects were blinded. A 4-point rating system was used to assess the degree of self-reported discomfort, such as stinging, tingling, itching, tightness, burning, or pain at the application site (0 = none, 1 = mild, 2 = moderate, and 3 = severe).

### 4.3. Biophysical Parameters Measurements

In July 2023, the measurements were completed. The subjects were told not to wash their face with any soaps, surfactants, or skin care products for at least 12 h before the study. The subjects then washed their faces under running water and gently dried them with paper towel before acclimating for 30 min in a room with controlled conditions (temperature of 22 °C and relative humidity of 50 ± 5%). The cheek (random side) and forehead were the study target areas. The forehead was used for testing skin sebum, while the cheeks were used for all other tests. To monitor skin biophysical parameters, probes were affixed to the MPA10 multiprobe adapter system (Courage and Khazaka, Cologne, Germany) and linked to a PC. The Corneometer CM825 (Courage and Khazaka, Cologne, Germany) was used to measure the moisture of the skin. The Tewameter TM 300 (Courage and Khazaka, Cologne, Germany) was used to measure TEWL, which represents skin barrier function. Facial sebum content was measured with the Sebumeter SM 815 (Courage and Khazaka, Cologne, Germany). The skin EI was measured by an MX18 Mexameter (Courage and Khazaka, Cologne, Germany). The skin redness a* value, based on the L*a*b* color system, was evaluated by a Colorimeter CL400 (Courage and Khazaka, Cologne, Germany). Each measurement was repeated at least three times, and the average readings were taken.

### 4.4. Skin Metabolites Sampling Process

D-squame (Dallas, TX, USA) was used to obtain skin samples from the cheek, which was the opposite side of taking probe measurements. D-squame was quickly extracted with forceps after being applied for 5 s at a circular pressure of 200 g by counterweight. After, the tape was peeled off, turned 90 degrees, and applied by counterweight once more for 5 s. The initial strip was thrown away. Twelve successive D-squame-stripped samples were then deposited into 1.5 mL reaction tubes, one for each of the two layers. D-squame tape strips were used to collect skin metabolites from the cheeks. The same individual carried out every step of the collecting process. D-squame samples were collected, snap-frozen in liquid nitrogen, and kept at −80 °C until tested. Samples were individually grounded with liquid nitrogen and the homogenate was resuspended with prechilled 80% methanol by well vortex. The samples were incubated on ice for 5 min and then were centrifuged at 15,000× *g*, 4 °C for 20 min. Some of supernatant was diluted to final concentration containing 53% methanol by LC-MS grade water. The samples were subsequently transferred to a fresh Eppendorf tube and then were centrifuged at 15,000× *g*, 4 °C for 20 min. Finally, the supernatant was injected into the LC-MS/MS system analysis [72].

### 4.5. UHPLC-MS/MS Analysis

UHPLC-MS/MS analyses were performed using a Thermo Syncronis C18 (2.1 mm × 100 mm, 1.7 µm) (Thermo Fisher Scientific, Germaring, Germany) UHPLC system (Thermo Fisher Scientific, Germaring, Germany) coupled with an Orbitrap Q Exactive^TM^ series mass spectrometer (Thermo Fisher Scientific, Germaring, Germany) in Scale Co., Ltd. (Beijing, China). Samples were injected onto a Hypesil Gold column (2.1 mm × 100 mm, 1.7 µm) (Thermo Fisher Scientific, Germaring, Germany) using an 18 min linear gradient at a flow rate of 0.2 mL/min. The eluents A and B were 0.1% FA in water and acetonitrile, respectively. The solvent gradient was set as follows: 0–1 min, 95% A, 1–5 min, 95–40% A, 5–8 min, 40–0%A, 8–11 min, 0%A, 11–14 min, 0–40%A, 11–15 min, 40–95% A, 15–18 min, 95% A. Q Exactive^TM^ series mass spectrometer was operated in positive/negative polarity mode with spray voltage of 3.2 kV, capillary temperature of 320 °C, sheath gas flow rate of 40 arb, and aux gas flow rate of 10 arb.

### 4.6. Data Processing and Metabolite Identification

The raw data files generated by UHPLC-MS/MS were processed using TraceFinder 3.2.0 (Thermo Fisher Scientific, Germaring, Germany) to perform peak alignment, peak picking, and quantitation for each metabolite. The main parameters were set as follows: retention time tolerance of 0.2 min, actual mass tolerance of 5 ppm, signal/noise ratio of 3, and minimum intensity. After that, peak intensities were normalized to the total spectral intensity. And then peaks were matched with the mzCloud (https://www.mzcloud.org/ accessed on 2 August 2024) and self-built database to obtain accurate qualitative and relative quantitative results. Statistical analyses were performed using the statistical software R (R version R-3.4.3), Python (Python 2.7.6 version), and CentOS (CentOS release 6.6). When data were not normally distributed, normal transformations were attempted by area normalization method.

### 4.7. Data Analysis

These metabolites were annotated using the KEGG database (https://www.genome.jp/kegg/pathway.html accessed on 3 August 2024), HMDB database (https://hmdb.ca/metabolites/ accessed on 3 August 2024), and LIPIDMaps database (http://www.lipidmaps.org/ accessed on 3 August 2024). Partial least squares discriminant analysis (PLS-DA) was performed at metaX (a flexible and comprehensive software for processing metabolomics data) [73]. We applied univariate analysis (*t*-test) to calculate the statistical significance (*p*-value). The metabolites with VIP > 1, *p*-value < 0.05, and fold change ≥ 2 or FC ≤ 0.5 were considered to be differential metabolites. Volcano plots were used to filter metabolites of interest based on log2 (fold change) and log10 (*p*-value) of metabolites by ggplot2 in R language. The differences in metabolite levels between the four groups were analyzed using the k-means algorithm, which is a distance-based clustering method based on the idea that the data are divided into K clusters such that the sum of the distances from each data point to the center of the cluster to which it belongs is minimized. We calculated the mean value of the substance in each group, which represents the expression level of the substance in the group, using the timeclust function of the “TCseq” package in R to perform k-means analysis on the metabolite expression data. The analysis parameters algo = “cm”, k = 12, standardize = TRUE, and other parameters were default. The cluster data with different expression patterns were finally obtained.

## 5. Conclusions

In conclusion, our results showed a substantial rise in sebum content, EI, a* values, TEWL, and LAST scores in the faces of participants in the OSS group as compared to the HS group, while their hydration decreased. This may be connected to the impaired skin barrier, which causes the stratum corneum of the skin to become dehydrated and the sebaceous glands to produce an excessive amount of oil. In addition, differences in skin metabolites between the OSS and HS groups were detected, particularly for lipids and lipid-like molecules as well as organic acids and derivatives. Furthermore, sphingolipid metabolism and amino acid metabolism were shown to be most disrupted in participants with OSS according to untargeted metabolomics screening. Our research indicates that the process of influencing corneocyte metabolism by changing sphingolipid and amino acid levels may be this target. The current study shows that skin barrier damage is present in OSS and also offers a target at the metabolic level to screen for skin barrier damage or even skin inflammation.

## Figures and Tables

**Table 1:** Classification of skin type, mean age, and scale score in subjects.

	Classification of Skin Types *n* (%)	Mean Age of Skin Types (Mean ± SD)	Scale Score with Sensitive Skin (Mean ± SD)	Scale Score with Oily Skin (Mean ± SD)
Total number	47 (100%)	32.55 ± 5.06	18.38 ± 11.93	23.02 ± 10.58
Healthy skin	12 (25.5%)	30.25 ± 5.19	2.25 ± 1.36	8.33 ± 3.70
Mild oily sensitive skin	12 (25.5%)	35.42 ± 4.24	14.58 ± 1.83 ***	20.00 ± 1.54 ***
Moderate oily sensitive skin	12 (25.5%)	31.25 ± 4.53	23.75 ± 1.06 ***	29.33 ± 1.87 ***
Severe oily sensitive skin	11 (23.4%)	33.36 ± 5.28	34.27 ± 2.41 ***	35.45 ± 2.46 ***

*** *p* < 0.001 compared with healthy skin.

**Table 2:** Overview of differential expression related to metabolic substances.

Compared Pairs	Ident Metabolite	Total Regulated	Up Regulated	Down Regulated
A vs. B	887	69	49	20
A vs. C	887	128	88	40
A vs. D	887	125	67	58
B vs. C	887	84	34	50
C vs. D	887	62	14	48
B vs. D	887	91	27	64

Note: A, healthy skin group, B, mild OSS group, C, moderate OSS group, D, severe OSS group.

## Data Availability

Data will be made available on request.

## Supplementary Materials

The following supporting information can be downloaded at: https://www.mdpi.com/article/10.3390/ijms252011033/s1.
