# Supplementary material for: Unveiling the Metabolomic Profile of Oily Sensitive Skin: A Non-Invasive Approach
Source: Int J Mol Sci. 2024 Oct 14;25(20):11033. doi: 10.3390/ijms252011033 (PMC11507585; doi:10.3390/ijms252011033)
Supplement: Supplementary file 1 [file ijms-25-11033-s001.zip › Figures S1-S6.pdf]

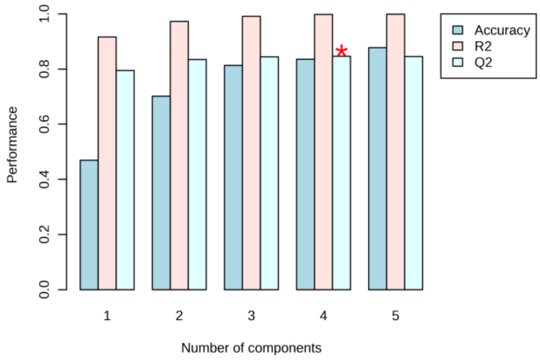

Figure S1. Cross validation of the developed PLS-DA models.

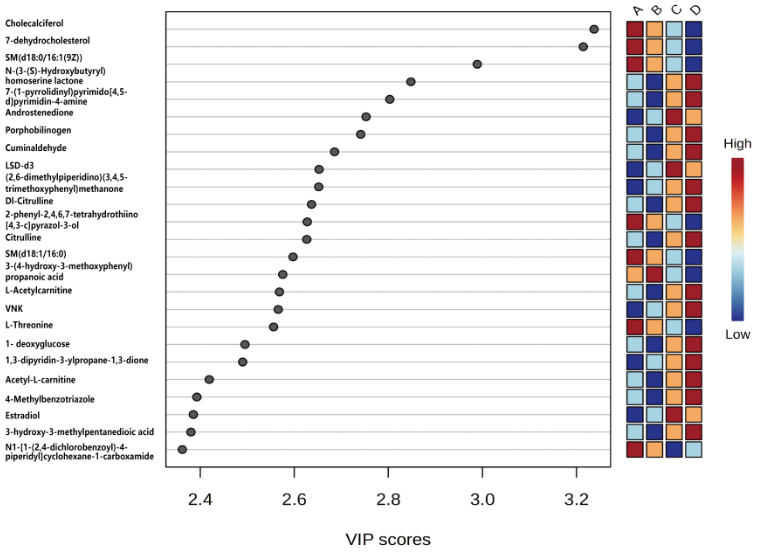

Figure S2. VIP scores of PLS-DA (A, healthy group, B, mild oily sensitive skin group, C, moderate oily sensitive skin group, D, severe oily sensitive skin group).

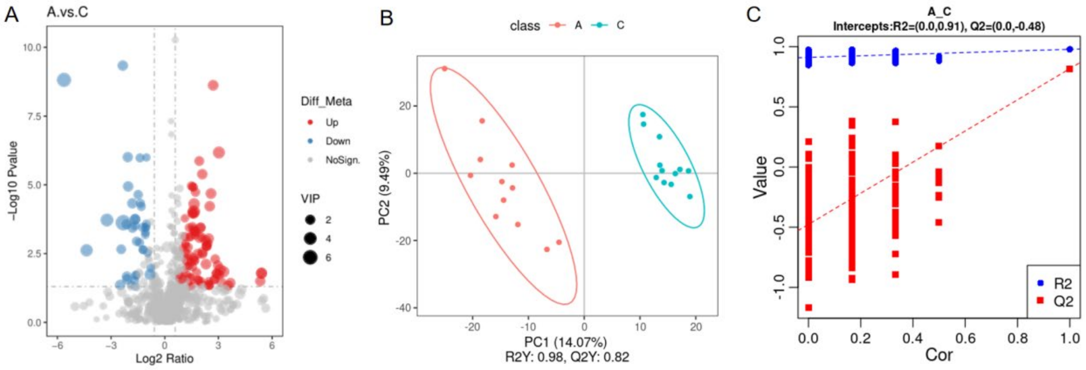

Figure S3. The results in subjects of metabolic volcano plots (A), PLS-DA plots (B) and validation plots (C) (A, healthy group, C, moderate oily sensitive skin group).

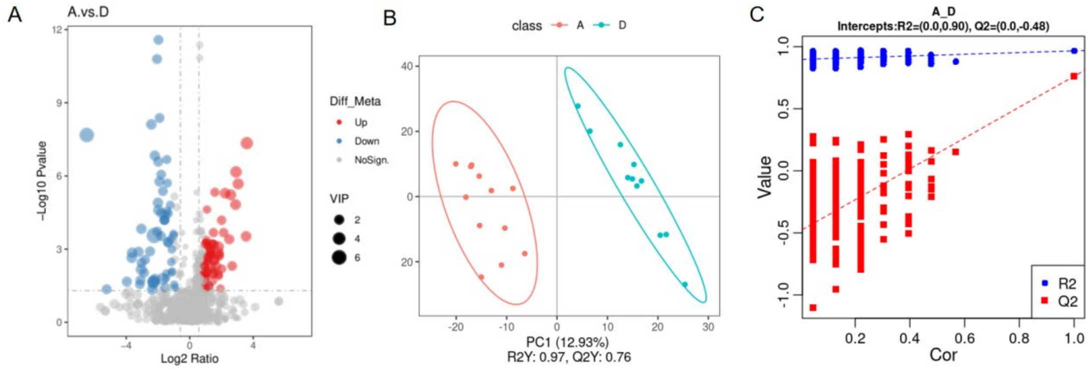

Figure S4. The results in subjects of metabolic volcano plots (A), PLS-DA plots (B) and validation plots (C) (A, healthy group, D, severe oily sensitive skin group).

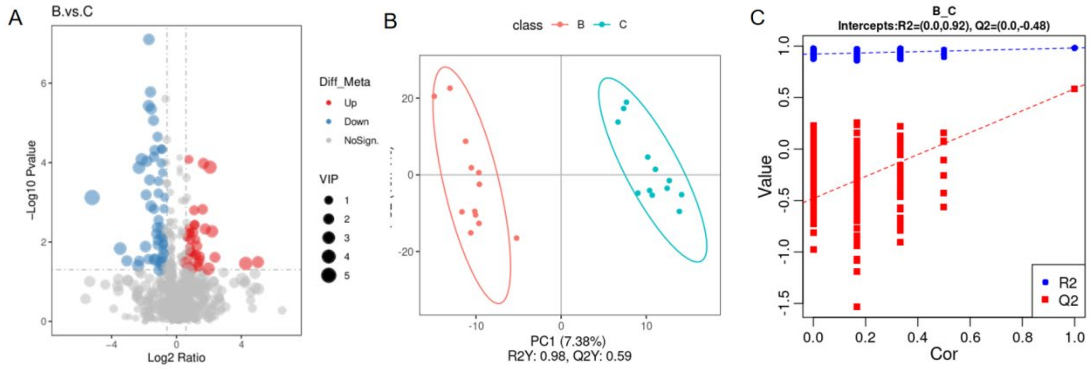

Figure S5. The results in subjects of metabolic volcano plots (A), PLS-DA plots (B) and validation plots (C) (B, mild oily sensitive skin group, C, moderate oily sensitive skin group).

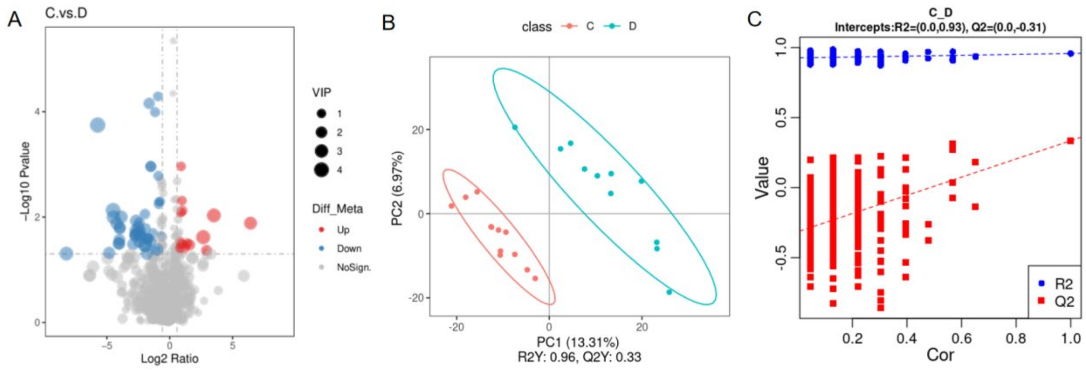

Figure S6. The results in subjects of metabolic volcano plots (A), PLS-DA plots (B) and validation plots (C) (C, moderate oily sensitive skin group, D, severe oily sensitive skin group).
